# Comparing the Efficacy of Carboplatin plus 5-Fluorouracil, Cisplatin plus 5-Fluorouracil, and Best Supportive Care for Advanced Esophageal Squamous Cell Carcinoma: A Propensity Score Analysis from a Tertiary Hospital in Southern Thailand

**DOI:** 10.3390/jcm13061735

**Published:** 2024-03-17

**Authors:** Jirapat Wonglhow, Panu Wetwittayakhlang, Patrapim Sunpaweravong, Chirawadee Sathitruangsak, Arunee Dechaphunkul

**Affiliations:** 1 Division of Medical Oncology, Department of Internal Medicine, Faculty of Medicine, Prince of Songkla University, Songkhla 90110, Thailand; jirapat.jw@gmail.com (J.W.); spatrapi@medicine.psu.ac.th (P.S.); sjirawadee@gmail.com (C.S.); 2 Division of Gastroenterology and Hepatology, Department of Internal Medicine, Faculty of Medicine, Prince of Songkla University, Songkhla 90110, Thailand; wet.panu@gmail.com

**Keywords:** esophageal cancer, squamous cell carcinoma, advanced stage, chemotherapy, carboplatin, 5-fluorouracil, cisplatin, best supportive care

## Abstract

**Background**: Although cisplatin plus 5-fluorouracil (5-FU) is the standard first-line treatment for advanced-stage esophageal squamous cell carcinoma (ESCC), carboplatin was substituted for cisplatin in cisplatin-ineligible patients. The efficacy of carboplatin plus 5-FU for advanced-stage ESCC remains unreported. **Methods**: This retrospective study analyzed first-line treatment—carboplatin plus 5-FU, cisplatin plus 5-FU, or best supportive care (BSC)—in advanced-stage ESCC patients at a tertiary hospital in Thailand (2012–2022). Survival was assessed using the Kaplan–Meier method, compared via the log-rank test, and adjusted through propensity score matching. Significance was set at *p* < 0.05. **Results**: Of 256 patients, 39.9% received carboplatin plus 5-FU, 27.7% cisplatin plus 5-FU, and 32.4% BSC. Carboplatin was significantly associated with older age, poorer performance status, more comorbidities, chronic kidney disease, and lower creatinine clearance. Median overall survival (OS) for carboplatin plus 5-FU, cisplatin plus 5-FU, and BSC was 8.05 (HR 0.31 [0.23, 0.43] vs. BSC, *p* < 0.001; HR 1.06 [0.78, 1.44] vs. cisplatin plus 5-FU, *p* = 0.7), 8.43, and 3.64 months, respectively. No significant OS difference was observed between carboplatin and cisplatin treatments after propensity score matching. Median progression-free survival (PFS) and objective response rates (ORR) showed no significant difference between carboplatin and cisplatin treatments. **Conclusions**: Despite less favorable baseline characteristics of patients receiving carboplatin plus 5-FU, this combination exhibited comparable OS, PFS, and ORR to cisplatin plus 5-FU in real-world scenarios. Furthermore, it significantly improved OS over BSC. Consequently, carboplatin plus 5-FU should be considered as an alternative regimen, particularly for advanced-stage ESCC patients who are ineligible for cisplatin.

## 1. Introduction

Esophageal cancer was diagnosed in approximately 604,100 cases globally in 2020, up from 450,000 cases in 2012 [1,2]. Notably, it ranks as the seventh most common cancer worldwide. Unfortunately, there has been little development in the treatment of esophageal cancer in the last two decades [3,4], and it holds the sixth leading cause of cancer-related deaths worldwide. In 2020, 544,000 deaths resulted from esophageal cancer, which reflects an increase from 400,000 cases in 2012 [1,2].

Esophageal cancer has two main histological subtypes: esophageal adenocarcinoma (EAC) and esophageal squamous cell carcinoma (ESCC). In Asia and Africa, ESCC remains the most common form of this cancer, whereas in Europe and the United States, EAC is predominant. In the most susceptible area, known as the esophageal cancer belt, spanning from northern Iran across Central Asia to the north-central regions of China, approximately 90% of individuals diagnosed with esophageal cancer exhibit ESCC [3,5]. At the time of diagnosis, approximately 50–60% of patients are unsuitable candidates for surgery [5,6]. This may be because of various reasons, such as tumor growth into adjacent structures, extensive lymph node metastases in the upper mediastinal or cervical regions, the presence of distant metastases, or the patient’s inability to undergo surgery because of medical conditions or poor performance status (PS). For most patients with unresectable or metastatic esophageal cancer, the primary focus of treatment is effective palliative care aimed at enhancing quality of life and extending survival.

In the context of metastatic or recurrent ESCC, the availability of effective and well-tolerated palliative chemotherapy options is limited, and phase III studies in this area are lacking [7]. Despite advancements in chemotherapy over the past three decades, individuals with metastatic ESCC typically experience a median overall survival (OS) of only around 8–11 months with combination chemotherapy [3,4,8,9]. In contrast to EAC, where survival benefits of targeted therapy for HER2 overexpressive tumors are observed, successful targeted therapy for ESCC has not yet been achieved [10,11,12]. However, with recent developments, a breakthrough in the use of immunotherapy in combination with chemotherapy has led to an OS prolongation of approximately 15 months [4,13,14,15]. Unfortunately, access to immunotherapy in Thailand remains limited because of its high cost. Among chemotherapeutic agents, cisplatin is one of the most effective, with a consistent single-agent response rate (RR) of 10–20% [8,16]. The combination of cisplatin and 5-fluorouracil (5-FU) has been extensively studied and is the most frequently used regimen in patients with esophageal cancer. The reported RRs for these two-drug combinations vary from 20% to 50% [5,8,16,17]. Nevertheless, cisplatin is known to cause renal toxicity, high-frequency sensorineural hearing loss, emesis, and peripheral neuropathy [18]. Hence, alternative chemotherapy regimens have been used, particularly in patients who are ineligible for cisplatin.

The REAL-2 trial, a phase III study, demonstrated the comparable efficacy of oxaliplatin to cisplatin in a three-drug combination regimen for patients with advanced esophagogastric cancer who had not received prior treatment, achieving an RR of 40–47% [19]. However, only approximately 10% of patients with ESCC were included in the REAL-2 trial. Carboplatin, another platinum analog, has shown limited effectiveness as a single-agent therapy for esophageal cancer [8,20]. Nevertheless, the results from phase II and retrospective trials evaluating carboplatin in combination with taxanes have shown encouraging outcomes [21,22,23]. Additionally, the combination of carboplatin and paclitaxel coupled with concurrent radiation therapy (RT) has become an internationally recognized standard of care for locally advanced diseases [17,24,25]. The efficacy of carboplatin and 5-FU concurrent with RT is comparable to that of cisplatin and 5-FU [26]. Taken together, carboplatin, when used in combination treatment, could be considered an alternative option for individuals ineligible for cisplatin-based therapy.

At our institution, carboplatin plus 5-FU has been used in patients with advanced ESCC who are not eligible for a cisplatin-based regimen. To the best of our knowledge, no information regarding the effectiveness of carboplatin plus 5-FU for the treatment of advanced or metastatic ESCC has been reported. Therefore, this study is the first to aim at presenting the real-world efficacy of first-line palliative chemotherapy with carboplatin plus 5-FU versus cisplatin plus 5-FU and best supportive care (BSC) for patients with advanced-stage ESCC at the Medical Oncology Service of a tertiary-care hospital in Southern Thailand.

## 2. Materials and Methods

### 2.1. Study Participants

From January 2012 to December 2022, we retrospectively reviewed the medical information of patients newly diagnosed with ESCC at Songklanagarind Hospital, Prince of Songkla University. The main inclusion criteria were as follows: (1) patients with histologically confirmed squamous cell carcinoma of the esophagus; (2) patients with advanced-stage disease according to the definition of clinical stage IV (cT4NxMx, cTxN3Mx, or cTxNxM1) according to the 8th edition of the American Joint Committee on Cancer (AJCC); (3) patients receiving first-line palliative chemotherapy as carboplatin plus 5-FU or cisplatin plus 5-FU with or without concurrent RT or BSC; and (4) patients aged 18 years or older. The main exclusion criterion was receiving first-line palliative chemotherapy with other regimens. 

Patient information was obtained from electronic medical records using the hospital information system of Songklanagarind Hospital. Information on initial clinical characteristics, including age at diagnosis, sex, body mass index (BMI), Eastern Cooperative Oncology Group (ECOG) PS, smoking and alcohol drinking status, comorbidities, including previous cancer and other concurrent primary cancers, tumor location, TNM staging, tumor differentiation from histopathology, site of metastasis, baseline laboratory data, and previous treatments, including surgery, concurrent chemoradiotherapy (CCRT), and palliative RT, was collected.

This study was reviewed and approved by the Ethics Committee of the Research Center of the Faculty of Medicine, Prince of Songkla University (REC.66422141). The requirement for written informed consent was waived because of the retrospective nature of the study. To ensure patient safety, all identifying information was excluded from the study.

### 2.2. Study Procedures

The chemotherapy regimen using carboplatin plus 5-FU was administered as follows: carboplatin was infused at an AUC of 5 over 1 h on day 1. Subsequently, a 24 h intravenous infusion of 5-FU was administered at a dose of 800 mg/m^2^ on days 1–5 or a dose of 1000 mg/m^2^ on days 1–4. The cisplatin plus 5-FU regimen involved the infusion of cisplatin at a dose of 80 or 100 mg/m^2^ over 1 h on day 1, followed by 5-FU administration in a manner similar to that of the carboplatin plus 5-FU regimen. Both chemotherapy regimens were repeated at 4-week intervals and continued for a maximum of four or six cycles, or until disease progression, death, onset of intolerable side effects, or upon the patient’s indication of preference. The chemotherapy dose was adjusted by primary oncologists based on the patient’s ECOG PS and baseline laboratory values.

If the first-line chemotherapy regimen was ineffective, subsequent therapeutic protocols were considered. The decision was made based on the patient’s performance status, personal preference, or the feasibility of using alternative agents.

### 2.3. Measurement

The primary objective of this study was to compare the OS of carboplatin plus 5-FU as first-line palliative chemotherapy with that of cisplatin plus 5-FU and BSC for advanced-stage ESCC. The secondary objectives were to compare OS using propensity score matching between carboplatin plus 5-FU and cisplatin plus 5-FU, progression-free survival (PFS), and objective response rate (ORR) between carboplatin plus 5-FU and cisplatin plus 5-FU. The prognostic factors for OS were also determined as secondary objectives. OS was defined as the period from the date of diagnosis of advanced-stage ESCC to death from any cause. PFS was defined as the period from the date of diagnosis to either radiologically identified tumor progression or death, whichever occurred first. Death status was validated and crosschecked using the Thai Social Security Death Index database. RR was evaluated using the Response Evaluation Criteria for Solid Tumors 1.1 criteria. Chest and abdominal computed tomography were performed every 2–3 months to elucidate treatment responses. RRs were determined for all patients (intention-to-treat analysis) and for those with assessable data.

### 2.4. Statistical Analysis

The sample size was calculated for survival based on a two-sample test to assess a difference in disease hazard (unequal sample) from the previous study [25], which evaluated carboplatin plus 5-FU concurrent with RT in localized-stage ESCC, as there was no previous study reporting the efficacy of carboplatin plus 5-FU in advanced-stage ESCC. A significance level (alpha) of 5% and a power level (beta) of 20% with a 10% allowance for missing data were used, resulting in a total requirement of 94 patients.

In terms of baseline characteristics, continuous variables are presented as median and interquartile range or as mean and standard deviation, where appropriate. Categorical variables were presented as frequencies and corresponding percentages. The Kaplan–Meier method was used to generate survival curves, which were compared using the log-rank test. The propensity score-matching principle with a 1:1 matching approach employing a nearest neighbor algorithm without replacement sampling was implemented for age, sex, ECOG PS, comorbidity, BMI, baseline creatinine clearance (CrCl), and metastatic status. The baseline characteristics of the patients were reassessed after matching. R software version 3.3.2 (R Foundation, Vienna, Austria) was used to perform statistical analyses. All *p*-values were two-sided, and statistical significance was set at *p* = 0.05.

## 3. Results

### 3.1. Baseline Characteristics

In total, 256 patients with advanced-stage ESCC were included in this study. Of these patients, 102, 71, and 83 received first-line palliative chemotherapy with carboplatin plus 5-FU (39.9%), cisplatin plus 5-FU (27.7%), or BSC (32.4%), respectively. The baseline characteristics of the patients are shown in Table 1. Compared with cisplatin plus 5-FU, patients receiving carboplatin plus 5-FU were associated with older age, poorer ECOG PS, more comorbidities, especially hypertension, cerebrovascular disease, chronic kidney disease, more histologically well-differentiated SCC, lower baseline hemoglobin and CrCl levels, and more palliative RT prior to palliative chemotherapy. Other factors were not significantly different between the groups.

### 3.2. Treatment Information

The median number of chemotherapy cycles was three and four cycles in carboplatin plus 5-FU and cisplatin plus 5-FU, respectively. The median dose of carboplatin in the carboplatin plus 5-FU group was AUC 5, and the median dose of cisplatin in the cisplatin plus 5-FU group was 80 mg/m^2^.

In both groups, the median 5-FU dose was 4000 mg/m^2^/cycle. Notably, patients in the carboplatin plus 5-FU group experienced a higher rate of chemotherapy dose reduction than did those in the cisplatin plus 5-FU group. CCRT was more prevalent in the cisplatin plus 5-FU group (71.8% vs. 46.1%; *p* = 0.001), with a median radiation dose of 5040 Gy.

Patients in the carboplatin plus 5-FU group most commonly discontinued treatment because of disease progression, followed by death and completion of the full treatment course. In contrast, patients in the cisplatin plus 5-FU group tended to discontinue treatment after completing the full treatment course, which was followed by disease progression and death. Approximately one-fourth of all patients received subsequent treatment (Table 2, Supplementary Table S1).

### 3.3. Effectiveness

#### 3.3.1. OS between Carboplatin plus 5-FU, Cisplatin plus 5-FU, and BSC

**Figure 1:**
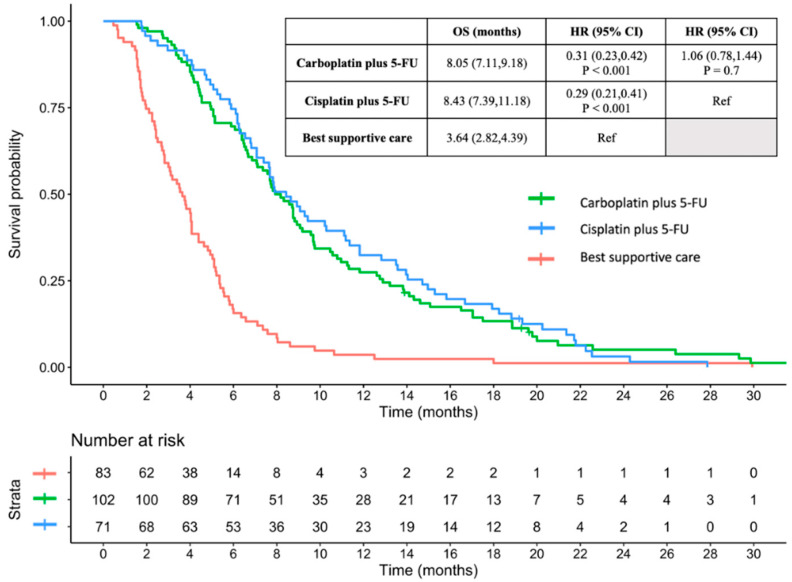
Overall survival between carboplatin plus 5-FU, cisplatin plus 5-FU, and best supportive care. OS: Overall survival; HR: hazard ratio; CI: confidence interval; 5-FU: 5-fluorouracil.

The median follow-up was 6.39 months. The median OS was 8.05 months in patients treated with carboplatin plus 5-FU (hazard ratio [HR] 0.31, 95% confidence interval [CI] 0.23–0.43, compared to that of BSC, *p* < 0.001; HR 1.06, 95% CI 0.78–1.44, compared to that of cisplatin plus 5-FU, *p* = 0.7), 8.43 months in those treated with cisplatin plus 5-FU (HR 0.29, 95% CI 0.21–0.41, compared to that of BSC, *p* < 0.001), and 3.64 months in those treated with BSC (Figure 1). The 1-year OS rates for patients treated with carboplatin plus 5-FU and cisplatin plus 5-FU were 27.5% and 32.4, respectively (HR, 1.14; *p* = 0.468).

#### 3.3.2. OS between Carboplatin plus 5-FU and Cisplatin plus 5-FU with Propensity Score Matching

**Figure 2:**
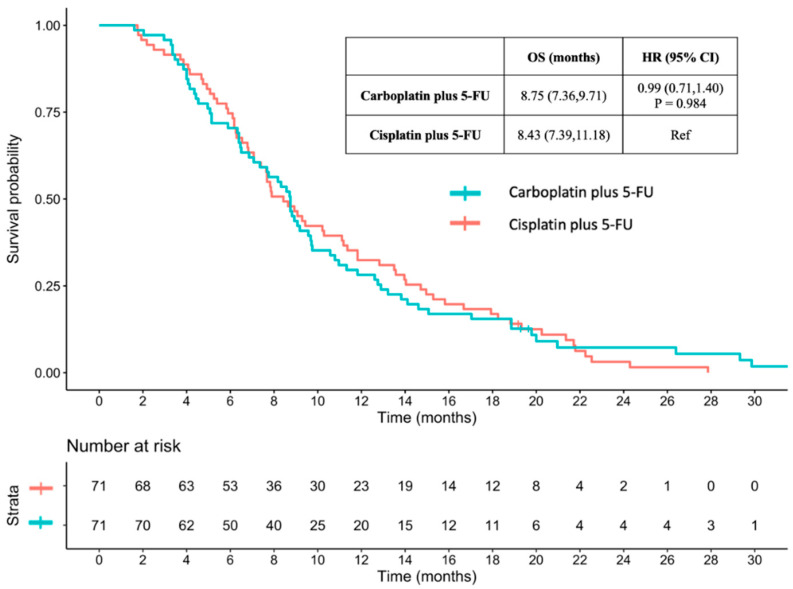
Overall survival between carboplatin plus 5-FU and cisplatin plus 5-FU with propensity score matching. OS: overall survival; HR: hazard ratio; CI: confidence interval; 5-FU: 5-fluorouracil.

After applying propensity score matching with age, sex, ECOG PS, comorbidity, BMI, baseline CrCl, and metastatic status, at a 1:1 ratio, 71 patients in the carboplatin plus 5-FU and cisplatin plus 5-FU groups were assessed. The baseline characteristics of the patients were reassessed after matching, revealing balance between the groups (Supplementary Table S2). The median OS times were 8.75 and 8.43 months for patients treated with carboplatin plus 5-FU and cisplatin plus 5-FU, respectively (Figure 2). There was no statistically significant difference between groups (HR 0.99, 95% CI 0.71–1.40; *p* = 0.984).

#### 3.3.3. PFS between Carboplatin plus 5-FU and Cisplatin plus 5-FU

**Figure 3:**
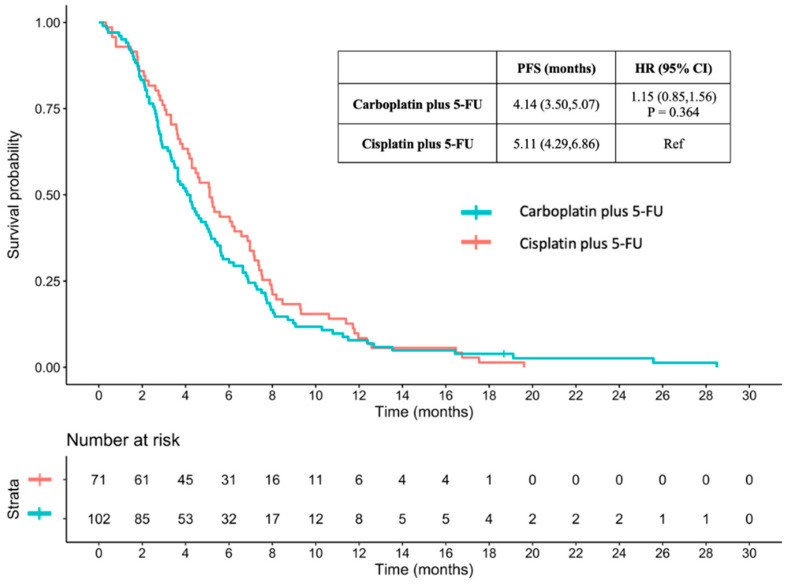
Progression-free survival between carboplatin plus 5-FU and cisplatin plus 5-FU. PFS: Progression-free survival; HR: hazard ratio; CI: confidence interval; 5-FU: 5-fluorouracil.

The median PFS for patients receiving carboplatin plus 5-FU and cisplatin plus 5-FU was 4.14 and 5.11 months (HR 1.15, 95% CI 0.85–1.56, *p* = 0.364), respectively (Figure 3).

Regarding the RR (Table 3), the ORR for carboplatin plus 5-FU and cisplatin plus 5-FU in the entire population was 30.4% and 36.6%, respectively (*p* = 0.436). Approximately one-fourth of the participants lacked a response evaluation. Thus, in patients with an available response assessment, the ORR was 41.9% and 47.3% for the carboplatin plus 5-FU and cisplatin plus 5-FU (*p* = 0.531), respectively.

### 3.4. Prognostic Factors

Various parameters were calculated using univariate Cox regression analysis to define the prognostic factors for OS. The significant factors were subsequently subjected to multivariate Cox regression analysis (Table 4). Patients who received palliative chemotherapy with either carboplatin or cisplatin plus 5-FU had significantly better OS when compared to those who received BSC alone. Patients with ECOG PS ≥ 2, presence of liver metastasis, presence of leukocytosis, declined CrCl (<60 mL/min), and hypoalbuminemia were significantly associated with poor OS.

## 4. Discussion

This study highlights the real-world efficacy of carboplatin plus 5-FU as first-line palliative chemotherapy in patients with advanced-stage ESCC. This regimen has been shown to provide outcomes comparable to the commonly used and standard regimen, cisplatin plus 5-FU. Furthermore, carboplatin plus 5-FU demonstrated superior outcomes compared to those of BSC alone.

The conventional first-line chemotherapy for advanced ESCC combines platinum and fluoropyrimidine [9,17,27,28]. Despite the majority of randomized trials focusing on esophageal adenocarcinoma, the overall findings are often extrapolated for application in ESCC [27]. Cisplatin plus 5-FU is the most apparent regimen for advanced-stage ESCC, although its benefits have not been confirmed in phase III clinical trials [29,30]. Nevertheless, several phase II studies have provided supportive data for cisplatin plus 5-FU, specifically in an ESCC population [16,31,32]. Hence, it has gained acceptance as a standard treatment regimen in various guidelines [17,27,33]. Nonetheless, cisplatin is known to cause renal toxicity, high-frequency sensorineural hearing loss, emesis, and peripheral neuropathy [18]. Therefore, alternative chemotherapy regimens have been used, particularly in patients who are ineligible for cisplatin.

Oxaliplatin, another platinum agent recommended as a substitute for cisplatin, has mostly been studied in adenocarcinoma [19]. Although previous studies have indicated an equivalence between cisplatin- and oxaliplatin-based regimens in patients with locoregionally advanced ESCC [34], oxaliplatin-containing regimens have been infrequently used in our institution because of reimbursement issues. In our practice, carboplatin can serve as an alternative agent in cisplatin-ineligible patients. The findings of the current study indicate that patients receiving carboplatin plus 5-FU demonstrated frailty markers, including older age, poorer ECOG PS, more comorbidities, lower baseline hemoglobin, and lower CrCl. These conditions represent real-world scenarios in which cisplatin is contraindicated.

In our study, the median OS for cisplatin plus 5-FU was 7.89 months, lower than that reported in recent studies of patients in the controlled arm of randomized controlled trials (RCTs) comparing cisplatin plus 5-FU with or without immunotherapy (9.8–10.7 months) [13,35]. This finding may be explained by the fact that our study, rooted in a real-world setting, comprised a population typically excluded from clinical trials because of increased fragility. Significantly, 13.7% of our patients receiving cisplatin plus 5-FU had ECOG PS 2, a subgroup not represented in any RCTs, and none in our study had ECOG PS 0, differing from RCTs where ECOG 0 accounted for approximately 40–48% [13,35]. Another factor contributing to the OS disparity was subsequent treatment, with only 25% of our study participants receiving subsequent systemic treatment compared to approximately 50% in RCTs [13,35]. Furthermore, real-world data from Japan reported a median OS of 10.4 months for cisplatin plus 5-FU, which is longer than that of our results, likely because of a higher percentage of patients with an ECOG PS of 0 (36.8%) and more patients receiving subsequent treatment (72.2%) [36]. PFS in our study was 5.1 months, comparable to that of other studies, ranging from approximately 3.6 to 5.8 months [13,32,35,36]. The ORR was also in line with previous reports, at 27.0–38.4% [13,31,35,36,37]. Taken together, our findings align with those of previous studies underscoring OS variations based on different baseline clinical characteristics and subsequent treatments.

Carboplatin plus 5-FU has not been extensively studied in advanced ESCC; however, our results suggest that this regimen could serve as a viable alternative when cisplatin is ineligible. Despite the retrospective study design, propensity score matching analysis confirmed a comparable OS between carboplatin plus 5-FU and cisplatin plus 5-FU, which is consistent with a previous study showing comparable efficacy of CCRT using carboplatin plus 5-FU and cisplatin plus 5-FU in localized-stage esophageal carcinoma [26].

Here, we also identified the prognostic factors for OS. Receiving palliative chemotherapy has emerged as a favorable prognostic factor, while leukocytosis, hypoalbuminemia, decreased CrCl, presence of liver metastasis, and poor ECOG PS were identified as unfavorable ones. Leukocytosis, an inflammatory signal crucial for cancer progression and metastasis, inhibits regulatory lymphocyte-T cell antitumor immunity and stimulates thrombosis, angiogenesis, and stromal remodeling [38]. Our results correlate with those of previous reports of poorer outcomes [39,40]. A diminished serum albumin level, beyond being a nutritional gauge, also serves as an inflammatory marker independently associated with poor survival [41]. Moreover, esophageal cancer particularly affects oral intake difficulty compared to other cancers, leading to cachexia and compromising survival, which is consistent with previous reports [42,43]. Renal dysfunction has also been reported to predict nephrotoxicity in patients receiving chemotherapy for esophageal cancer, which could affect survival [44]. Notably, the compromised kidney function impairs drug clearance and increases the risk of toxicity. This impairment also restricts the choice of chemotherapeutic drugs and challenges a patient’s tolerance to cancer treatments, potentially prompting modifications or dose reductions in chemotherapy regimens and impacting treatment effectiveness. Additionally, liver metastasis can result in complications such as liver failure, jaundice, and ascites, significantly affecting a patient’s quality of life and contributing to disease severity. Our study’s results align with Luo’s findings, in which the presence of liver metastasis led to shorter survival [45]. In the liver metastasis group, individuals with upper esophageal cancer had the worst survival rates, followed by those with middle- and lower-segment involvement [46].

A key strength of our study, based on the current knowledge, is that it is the first to examine the effectiveness of carboplatin plus 5-FU as a first-line palliative treatment for patients with advanced-stage ESCC. We implemented propensity score-matching analysis to mitigate selection bias. Our findings underscore that the OS, PFS, and ORR of the carboplatin plus 5-FU regimen were not significantly different from those of the cisplatin plus 5-FU regimen in a real-world context. Additionally, we identified the prognostic factors associated with OS in patients with advanced ESCC. Our findings can help guide physicians in tailoring treatment and decision-making processes for individuals.

However, it is essential to consider the limitations of this study; its retrospective nature led to missing data and a selection bias. Regarding the single-center design, the study’s sample size may be relatively small, warranting caution when generalizing the findings to other settings based on patient baseline characteristics.

In summary, despite the less favorable baseline characteristics of patients receiving carboplatin plus 5-FU, this combination demonstrated acceptable effectiveness in terms of OS, PFS, and ORR in real-world scenarios. No significant differences were observed when compared to individuals treated with cisplatin plus 5-FU, even after propensity score matching. Notably, carboplatin plus 5-FU significantly improved OS compared to the use of BSC. Consequently, it should be considered as an alternative regimen, particularly for patients with advanced-stage ESCC who are ineligible for cisplatin. However, additional studies are required to illustrate the efficacy of carboplatin plus 5-FU in a larger prospective cohort.

## Figures and Tables

**Table 1:** Baseline characteristics.

	Carboplatin Plus 5-FU (*n* = 102)	Cisplatin Plus 5-FU (*n* = 71)	Best Supportive Care (*n* = 83)
Median Age, years (IQR) *^,^**	63.0 (58.2, 68.6)	54.6 (49.9, 59.9)	62.1 (54.2, 71)
Age ≥ 65 years, *n* (%) *^,^**	40 (39.2)	5 (7.0)	37 (44.6)
Sex, *n* (%)			
Male	96 (94.1)	67 (94.4)	76 (91.6)
Female	6 (5.9)	4 (5.6)	7 (8.4)
ECOG PS, *n* (%) *^,^**			
0	2 (2)	0 (0)	0 (0)
1	70 (68.6)	61 (85.9)	11 (13.3)
2	29 (28.4)	10 (14.1)	48 (57.8)
3	1 (1)	0 (0)	22 (26.5)
4	0 (0)	0 (0)	2 (2.4)
BMI, *n* (%) **			
<18.5 kg/m^2^	59 (57.8)	40 (56.3)	63 (75.9)
18.5–22.9 kg/m^2^	33 (32.4)	23 (32.4)	19 (22.9)
23.0–24.9 kg/m^2^	5 (4.9)	5 (7.0)	1 (1.2)
≥25 kg/m^2^	5 (4.9)	3 (4.2)	0 (0)
Smoking, *n* (%) **			
Current or former	92 (90.2)	63 (88.7)	60 (72.3)
Never	10 (9.8)	8 (11.3)	23 (27.7)
Alcohol drinking, *n* (%) **			
Current or former	88 (86.3)	62 (87.3)	55 (66.3)
Never	14 (13.7)	9 (12.7)	28 (33.7)
Comorbidities, *n* (%) *^,^**	59 (57.8)	26 (36.6)	38 (45.8)
Hypertension *^,^**	35 (34.3)	11 (15.5)	26 (31.3)
Diabetes mellitus	7 (6.9)	4 (5.6)	9 (10.8)
Ischemic heart disease	3 (2.9)	1 (1.4)	5 (6)
Cerebrovascular disease	8 (7.8)	2 (2.8)	6 (7.2)
COPD	5 (4.9)	2 (2.8)	2 (2.4)
Cirrhosis	6 (5.9)	4 (5.6)	1 (1.2)
Chronic kidney disease *^,^**	78 (76.5)	43 (60.6)	75 (90.4)
History of previous cancer, *n* (%)	11 (10.8)	2 (2.8)	5 (6)
Concurrent second primary cancer, *n* (%) **	3 (2.9)	7 (9.9)	0 (0)
Tumor Location, *n* (%)			
Cervical	7 (6.9)	10 (14.1)	2 (2.4)
Upper thoracic	23 (22.5)	10 (14.1)	16 (19.3)
Middle thoracic	53 (52)	28 (39.4)	42 (50.6)
Lower thoracic	17 (16.7)	21 (29.6)	20 (24.1)
Esophagogastric junction	2 (2)	2 (2.8)	3 (3.6)
T stage, *n* (%)			
T1	0 (0)	0 (0)	1 (1.2)
T2	5 (4.9)	4 (5.6)	6 (7.2)
T3	48 (47.1)	25 (35.2)	37 (44.6)
T4	49 (48)	42 (59.2)	39 (47)
N stage, *n* (%)			
N0	5 (4.9)	2 (2.8)	5 (6)
N1	56 (54.9)	42 (59.2)	45 (54.2)
N2	22 (21.6)	17 (23.9)	21 (25.3)
N3	19 (18.6)	10 (14.1)	12 (14.5)
M stage, *n* (%) **			
M0	38 (37.3)	30 (42.3)	17 (20.5)
M1	64 (62.7)	41 (57.7)	66 (79.5)
Tumor differentiation, *n* (%) *			
Well differentiated SCC	23 (22.5)	8 (11.3)	15 (18.1)
Moderately differentiated SCC	44 (43.1)	43 (60.6)	41 (49.4)
Poorly differentiated SCC	25 (24.5)	18 (25.4)	19 (22.9)
Missing	10 (9.8)	2 (2.8)	8 (9.6)
Number of organ metastasis, *n* (%) **			
1	40 (39.2)	27 (38.0)	41 (50.0)
2	18 (17.6)	12 (16.9)	18 (22.0)
≥3	6 (5.9)	1 (1.4)	6 (7.5)
Organ metastasis, *n* (%)			
Lung	24 (23.5)	12 (16.9)	16 (19.3)
Lymph node **	28 (27.5)	25 (35.2)	41 (49.4)
Liver	20 (19.6)	13 (18.3)	21 (25.3)
Bone	10 (9.8)	4 (5.6)	12 (14.5)
Adrenal gland	2 (2.0)	1 (1.4)	1 (1.2)
Peritoneum	1 (1.0)	0 (0)	1 (1.2)
Pleura **	3 (2.9)	0 (0)	6 (7.2)
Others	4 (3.9)	0 (0)	0 (0)
Laboratory	8905 (7090, 11,527)	9530 (7650, 12,185)	10,020 (7700, 13,185)
White blood cell count, per µL (IQR)	10.8	11.7	10.6
Hemoglobin, g/dL (IQR) *^,^**	(9.8, 11.9)	(10.6, 12.9)	(9.3, 11.8)
Platelet count, per µL (IQR)	351,670 (124,937.5)	379,169 (147,677.9)	361,791.4 (133,515.2)
Creatinine, mg/dL (IQR)	0.8 (0.7, 0.9)	0.7 (0.6, 0.9)	0.8 (0.6, 1)
CrCl, mL/min (IQR) *^,^**	50.4 (45.2, 58.3)	57.5 (50.3, 65.3)	45.2 (39.5, 53.1)
CrCl < 60 mL/min, *n* (%) *^,^**	78 (76.5)	43 (60.6)	75 (90.4)
CrCl ≥ 60 mL/min, *n* (%) *^,^**	24 (23.5)	28 (39.4)	8 (9.6)
Albumin, g/dL (SD) **	3.5 (0.5)	3.7 (0.6)	3.2 (0.5)
Previous treatment, *n* (%)			
Esophagectomy	2 (2.0)	2 (2.8)	6 (7.2)
Definitive CCRT	2 (2.0)	0 (0)	0 (0)
Palliative RT *^,^**	27 (26.5)	5 (7.0)	38 (45.8)

* *p*-value significant (<0.05) between carboplatin plus 5-FU and cisplatin plus 5-FU; ** *p*-value significant (<0.05) among carboplatin plus 5-FU, cisplatin plus 5-FU, and best supportive care; 5-FU: 5-fluorouracil; IQR: interquartile range; ECOG: Eastern Cooperative Oncology Group; PS: performance status; BMI: body mass index; COPD: chronic obstructive pulmonary disease; SCC: squamous cell carcinoma; CrCl: creatinine clearance; SD: standard deviation; CCRT: concurrent chemoradiotherapy; RT: radiotherapy.

**Table 2:** Treatment information.

	Carboplatin Plus 5-FU (*n* = 102)	Cisplatin Plus 5-FU (*n* = 71)
Treatment		
Number of chemotherapy cycles (IQR)	3 (2, 4)	4 (2, 4)
Dose of carboplatin, AUC (IQR)	5 (4, 5)	-
Dose of cisplatin, mg/m^2^	-	80 (80, 80)
Dose of 5-FU, mg/m^2^/cycle	4000 (3200, 4000)	4000 (4000, 4000)
Any dose reduction, *n* (%)	55 (53.9)	21 (29.5)
One level	52 (51.0)	17 (23.9)
Two level	3 (2.9)	4 (5.6)
Concurrent RT, *n* (%) *	47 (46.1)	51 (71.8)
Dose of RT, Gray (IQR)	5040 (5000, 5040)	5040 (5000, 5040)
Discontinuation, *n* (%)		
Complete treatment	20 (19.6)	24 (33.8)
Progressive disease	35 (34.3)	19 (26.8)
Death	31 (30.4)	16 (22.5)
Worsening ECOG PS	10 (9.8)	8 (11.3)
Patient preference	5 (4.9)	2 (2.8)
Refer	0 (0)	2 (2.8)
Loss of follow-up	1 (1.0)	0 (0)
Subsequent therapy, *n* (%)	27 (26.5)	19 (26.8)
Second line treatment	27 (26.5)	19 (26.8)
Third line treatment	2 (2.0)	4 (5.6)
Fourth line treatment	1 (1.0)	0 (0)
Fifth line treatment	1 (1.0)	0 (0)

* *p*-value significant (*p* < 0.05); 5-FU, 5-fluorouracil; IQR, interquartile range; AUC, area under the curve; RT, radiotherapy; ECOG, Eastern Cooperative Oncology Group; PS, performance status.

**Table 3:** Response rate.

	Carboplatin Plus 5-FU (*n* = 102)	Cisplatin Plus 5-FU (*n* = 71)	*p*-Value
Evaluable, *n* (%)	74 (72.5)	55 (77.5)	
Complete response, *n* (%)	0 (0)	0 (0)	
Partial response, *n* (%)	31 (30.4)	26 (36.6)	
Stable disease, *n* (%)	21 (20.6)	15 (21.1)	
Progressive disease, *n* (%)	22 (21.6)	14 (19.7)	
Missing, *n* (%)	28 (27.5)	16 (22.5)	
ORR in the entire population, *n* (%)	31 (30.4)	26 (36.6)	0.436
ORR in available data, *n* (%)	31 (41.9)	26 (47.3)	0.531

5-FU, 5-fluorouracil; ORR, objective response rate.

**Table 4:** Prognostic factors for overall survival.

	Univariate Cox Regression Analysis		Multivariate Cox Regression Analysis	
	HR	95% CI	HR	95% CI
Receiving chemotherapy	0.31 *	0.23, 0.40	0.46 *	0.33, 0.64
Age ≥ 65 years	1.41 *	1.08, 1.84	1.16	0.85, 1.58
Sex–Male	1.23	0.74, 2.04		
ECOG PS ≥ 2	2.06 *	1.60, 2.66	1.43 *	1.06, 1.94
BMI < 18.5 kg/m^2^	1.27	0.98, 1.64		
Smoking	0.87	0.62, 1.22		
Alcohol drinking	0.79	0.58, 1.07		
History of previous cancer	0.89	0.55, 1.45		
Concurrent two primary cancer	1.51	0.80, 2.85		
Tumor Location				
Cervical	Ref			
Upper thoracic	1.25	0.74, 2.14		
Middle thoracic	1.18	0.73, 1.92		
Lower thoracic	1.27	0.75, 2.13		
Esophagogastric junction	1.17	0.49, 2.80		
T stage				
T1	Ref			
T2	0.12 *	0.02, 0.92	0.69	0.08, 5.57
T3	0.11 *	0.01, 0.81	0.44	0.06, 3.35
T4	0.10 *	0.01, 0.71	0.44	0.06, 3.40
N stage				
N0	Ref			
N1	1.03	0.56, 1.91		
N2	1.08	0.56, 2.06		
N3	0.99	0.51, 1.95		
M stage–M1	1.56 *	1.19, 2.03	1.21	0.86, 1.70
Tumor differentiation				
Well differentiated SCC	Ref			
Moderately differentiated SCC	1.19	0.85, 1.68		
Poorly differentiated SCC	1.08	0.73, 1.60		
Number of organ metastasis ≥ 2	1.01	0.79, 1.30		
Liver metastasis	1.57 *	1.16, 2.13	1.43 *	1.01, 2.03
White blood cell count > 10,000/uL	1.78 *	1.38, 2.30	1.86 *	1.38, 2.50
Hemoglobin < 10 g/dL	1.58 *	1.19, 2.09	1.06	0.77, 1.45
Platelet				
<150,000/uL	0.99	0.51, 1.96		
>450,000/uL	1.09	0.82, 1.46		
CrCl < 60 mL/min	1.50 *	1.12, 2.02	1.47 *	1.05, 2.05
Albumin < 3.5 g/dL	2.13 *	1.65, 2.76	1.48 *	1.09, 2.02

* *p*-value significant (*p* < 0.05). HR, hazard ratio; CI, confidence interval; ECOG, Eastern Cooperative Oncology Group; PS, performance status; BMI, body mass index; Ref, reference; SCC, squamous cell carcinoma; CrCl, creatinine clearance.

## Data Availability

The datasets used and/or analyzed during the current study are available from the corresponding author upon reasonable request.

## Supplementary Materials

The following supporting information can be downloaded at: https://www.mdpi.com/article/10.3390/jcm13061735/s1, Table S1: Baseline characteristics and treatment information; Table S2: Baseline characteristics after propensity score matching.
