# Supplementary material for: Comparing the Efficacy of Carboplatin plus 5-Fluorouracil, Cisplatin plus 5-Fluorouracil, and Best Supportive Care for Advanced Esophageal Squamous Cell Carcinoma: A Propensity Score Analysis from a Tertiary Hospital in Southern Thailand
Source: J Clin Med. 2024 Mar 17;13(6):1735. doi: 10.3390/jcm13061735 (PMC10971724; doi:10.3390/jcm13061735)
Supplement: Supplementary file 1 [file jcm-13-01735-s001.zip › jcm-2906600-supplementary.pdf]

Supplementary Table S1 Baseline characteristics and treatment information

	Carboplatin plus 5-FU (*n* = 102)	Cisplatin plus 5-FU (*n* = 71)	Best supportive care (*n* = 83)
History of previous cancer	11 (10.8)	2 (2.8)	5 (6)
Base of tongue	1 (1.0)	0 (0)	1 (1.2)
Floor of month	2 (2.0)	0 (0)	1 (1.2)
Cervical lymph node metastasis	0 (0)	0 (0)	1 (1.2)
Supraglottis	2 (2.0)	1 (1.4)	2 (2.4)
Glottis	1 (1.0)	1 (1.4)	0 (0)
Hypopharynx	2 (2.0)	0 (0)	0 (0)
Pyriformis	3 (2.9)	0 (0)	0 (0)
Tonsil	1 (1.0)	0 (0)	0 (0)
Lung	0 (0)	0 (0)	1 (1.2)
Concurrent two primary cancer	3 (2.9)	7 (9.9)	0 (0)
Base of tongue	0 (0)	1 (1.4)	0 (0)
Supraglottis	0 (0)	2 (2.8)	0 (0)
Soft palate	0 (0)	1 (1.4)	0 (0)
Hypopharynx	0 (0)	1 (1.4)	0 (0)
Pyriformis	1 (1.0)	2 (2.8)	0 (0)
Tonsil	2 (2.0)	0 (0)	0 (0)
Organ metastasis, n (%)			
Lung	24 (23.5)	12 (16.9)	16 (19.3)
Lymph node	28 (27.5)	25 (35.2)	41 (49.4)
Liver	20 (19.6)	13 (18.3)	21 (25.3)
Bone	10 (9.8)	4 (5.6)	12 (14.5)
Brain	1 (1.0)	0 (0)	0 (0)
Adrenal gland	2 (2.0)	1 (1.4)	1 (1.2)
Peritoneum	1 (1.0)	0 (0)	1 (1.2)
Pleura	3 (2.9)	0 (0)	6 (7.2)
Spleen	1 (1.0)	0 (0)	0 (0)
Kidney	1 (1.0)	0 (0)	0 (0)
Small bowel	1 (1.0)	0 (0)	0 (0)
Subsequent therapy	27 (26.5)	19 (26.8)	
Second line treatment	27 (26.5)	19 (26.8)	
Paclitaxel	23 (22.5)	14 (19.7)	
Pembrolizumab	1 (1.0)	1 (1.4)	
Clinical trial	3 (3.0)	3 (4.2)	
Carboplatin plus 5-FU	0 (0)	1 (1.4)	
Third line treatment	2 (2.0)	4 (5.6)	
Carboplatin plus etoposide	1 (1.0)	1 (1.4)	
Capecitabine plus oxaliplatin	0 (0)	1 (1.4)	

Paclitaxel	1 (1.0)	2 (2.8)	
Fourth line treatment	1 (1.0)	0 (0)	
Docetaxel	1 (1.0)	0 (0)	
Fifth line treatment	1 (1.0)	0 (0)	
Gemcitabine	1 (1.0)	0 (0)	

Supplementary Table S2 Baseline characteristics after propensity score matching

	Carboplatin plus 5-FU (*n* = 71)	Cisplatin plus 5-FU (*n* = 71)	P-value
Age ≥ 65 years, n (%)	10 (14.1)	5 (7.0)	0.275
Sex, n (%)			
Male	67 (94.4)	67 (94.4)	1.000
Female	4 (5.6)	4 (5.6)	
ECOG PS, n (%)			
0	0	0	
1	51 (71.8)	61 (85.9)	
2	20 (28.2)	10 (14.1)	0.064
3	0	0	
4	0	0	
Comorbidities, n (%)	35 (49.3)	26 (36.6)	0.175
BMI, n (%)			
< 18.5 kg/m^2	42 (59.2)	40 (56.4)	
18.5–22.9 kg/m^2	24 (33.8)	23 (32.4)	0.725
23.0–24.9 kg/m^2	2 (2.8)	5 (7.0)	
≥ 25 kg/m^2	3 (4.2)	3 (4.2)	
M stage, n (%)			
M0	28 (39.4)	30 (42.3)	0.864
M1	43 (60.6)	41 (57.7)	
CrCl			
< 60 mL/min , n (%)	50 (70.4)	43 (60.6)	0.29
≥ 60 mL/min, n (%)	21 (29.6)	28 (39.4)	

5-FU: 5-fluorouracil; ECOG: Eastern Cooperative Oncology Group; PS: performance status; BMI: body mass index; CrCl: creatinine clearance.
